# The relation between inflammatory biomarkers and drug pharmacokinetics in the critically ill patients: a scoping review

**DOI:** 10.1186/s13054-024-05150-4

**Published:** 2024-11-19

**Authors:** Letao Li, Julia Zinger, Sebastiaan D. T. Sassen, Nicole P. Juffermans, Birgit C. P. Koch, Henrik Endeman

**Affiliations:** 1 Department of Hospital Pharmacy, Erasmus MC-University Medical Center, Doctor Molewaterplein 40, 3015 GD Rotterdam, The Netherlands; 2 Department of Hospital Pharmacy, Xinqiao Hospital, Army Medical University, 183 Xinqiao Street, Shapingba District, Chongqing, 400037 China; 3 Department of Intensive Care, Erasmus MC-University Medical Center, Doctor Molewaterplein 40, 3015 GD Rotterdam, The Netherlands; 4 Department of Intensive Care, OLVG, Oosterpark 9, 1091 AC Amsterdam, The Netherlands

**Keywords:** Pharmacokinetics, Critically ill patient, Intensive care unit, Inflammation, Biomarker, CRP

## Abstract

**Background:**

The level of inflammation alters drug pharmacokinetics (PK) in critically ill patients. This might compromise treatment efficacy. Understanding the specific effects of inflammation, measured by biomarkers, on drug absorption, distribution, metabolism, and excretion is might help in optimizing dosing strategies.

**Objectives:**

This review investigates the relationship between inflammatory biomarkers and PK parameters absorption, distribution, metabolism and excretion (ADME) in critically ill patients, providing insight in the complexity of dosing drugs in critically ill patients.

**Method:**

Following PRISMA guidelines, we conducted a comprehensive search of Medline, Embase, Web of Science, and Cochrane databases (January 1946–November 2023). Studies examining inflammatory biomarkers, PK parameters, or drug exposure in critically ill patients were included. Records were screened by title, abstract, and full text, with any discrepancies resolved through discussion or consultation with a third reviewer.

**Results:**

Of the 4479 records screened, 31 met our inclusion criteria: 2 on absorption, 7 on distribution, 17 on metabolism, and 6 on excretion. In general, results are only available for a limited number of drugs, and most studies are done only looking at one of the components of ADME. Higher levels of inflammatory biomarkers may increase or decrease drug absorption depending on whether the drug undergoes hepatic first-pass elimination. For drug distribution, inflammation is negatively correlated with drug protein binding capacity, positively correlated with cerebrospinal fluid penetration, and negatively correlated with peritoneal penetration. Metabolizing capacity of most drugs was inversely correlated with inflammatory biomarkers. Regarding excretion, inflammation can lead to reduced drug clearance, except in the neonatal population.

**Conclusion:**

Inflammatory biomarkers can offer valuable information regarding altered PK in critically ill patients. Our findings emphasize the need to consider inflammation-driven PK variability when individualizing drug therapy in this setting, at the same time research is limited to certain drugs and needs further research, also including pharmacodynamics.

**Supplementary Information:**

The online version contains supplementary material available at 10.1186/s13054-024-05150-4.

## Introduction

Inflammation, a key characteristic of critical illness, significantly impacts drug pharmacokinetics (PK). This can lead to challenges in achieving optimal drug dosing, potentially resulting in both under- and overdosing. While clinicians routinely adjust drug doses based on organ function and drug interactions, the influence of inflammation is often overlooked. However, no review has comprehensively summarized the relationship between readily available inflammatory biomarkers and PK parameters in critically ill patients.

**Fig. 1:**
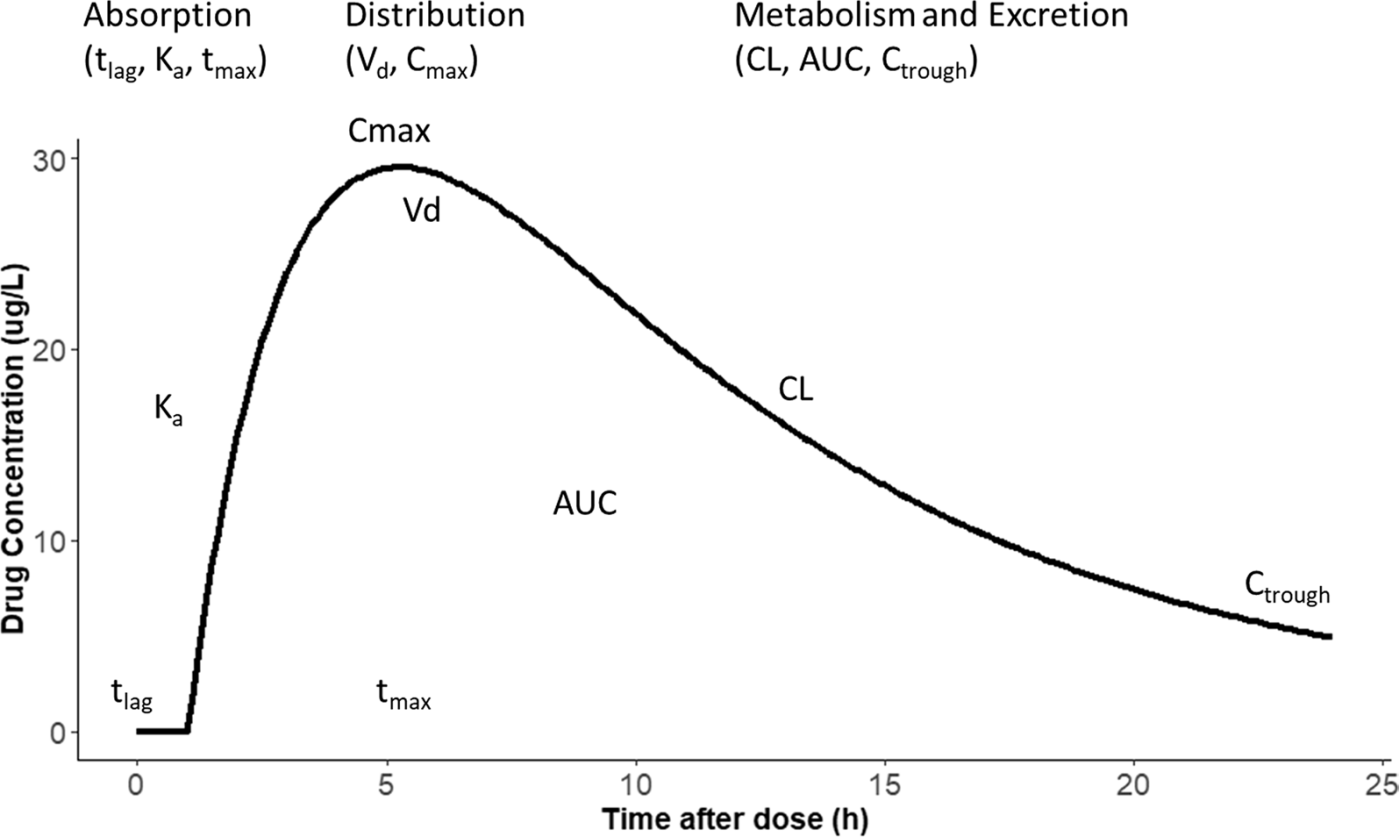
Brief examples about Effect of absorption, distribution, metabolism and extraction (ADME) related pharmacokinetic parameters on pharmacodynamics. t_lag_ (lag time): This is the time delay between the administration of a drug and its appearance in the systemic circulation. Ka (Absorption Rate Constant): The rate at which a drug leaves the site of administration and enters the systemic circulation. The higher the Ka, the faster the absorption. Vd (Volume of distribution): This parameter describes the extent to which a drug is distributed into body tissues relative to plasma. CL (clearance): The volume of plasma from which a drug is completely removed per unit time. t_max_: time to reach peak concentration. Cmax: maximum concentration. AUC: area under the curve. C_trough_: trough concentration

Absorption, distribution, metabolism, and excretion (ADME) processes govern drug behavior, this review simplifies these processes with key pharmacokinetic (PK) parameters as shown in Fig. 1. A thorough grasp of these PK parameters is essential for implementing precise dosing strategies in clinical practice. This is particularly critical in the context of critically ill patients. An example of this is optimization of antibiotic therapy, especially in relation to the increase in multi-drug resistant pathogens with elevated minimum inhibitory concentrations (MICs). Thus, exploring the factors that influence the intricate relationship between PK is paramount for optimizing dosing strategies and improving patient outcomes in this vulnerable population.

Although the concept of precision dosing has long been mentioned in clinical practice, the application of precision dosing in critically ill patients is still challenging due to the limited pharmacokinetic (PK) studies specifically conducted on critically ill patients. Drugs are prescribed at equivalent doses for both critically ill patients and those who are not critically ill. This practice is based on the assumption that the drug kinetics are comparable in these two groups, resulting in patients frequently receiving identical dosages within the same treatment cycle. However, many studies have shown that critically ill patients exhibit distinct PK parameters compared to those in non-ICU wards [1–5], compounded by their highly heterogeneity and dynamically evolving conditions [6–8]. These factors hinder the reliability of existing pharmacokinetic models in predicting drug concentrations in critically ill patients, underscoring the urgent need to identify variables that influence drug PK to enhance precision dosing in this patient group.

Inflammation, a complex biological response triggered by harmful stimuli [9], can escalate into a systemic inflammatory response syndrome (SIRS) in critically ill patients, impacting multiple organs and systems [10]. This systemic inflammation, often observed in conditions such as sepsis, is characterized by elevated levels of inflammatory markers like C-reactive protein (CRP), interleukin-6 (IL-6), and tumor necrosis factor-alpha (TNF-α). These biomarkers serve as crucial indicators for the development of complications such as multiple organ dysfunction syndrome (MODS) [11], and can also be used to predict and monitor the progression of diseases like acute respiratory distress syndrome (ARDS) and delirium in the ICU [12, 13].

Existing research has demonstrated that inflammation can trigger changes in a patient’s physiological state that might change the drug pharmacokinetics [14–19]. Recent studies in non-ICU wards have revealed a significant correlation between inflammatory status and drug PK [20–31]. Numerous studies have demonstrated a significant association between inflammatory biomarkers and the activity of hepatic cytochrome P450 (CYP) enzymes and membrane transporters, key components of drug metabolism and disposition [32–36]. Given that inflammation is more prevalent and more intense among critically ill patients-often triggered by infections, surgeries, severe burns, trauma, or tumors [37]-this relationship warrants closer examination in the critical care setting. The intensity of inflammation in the critically ill together with the frequent occurrence of organ failure renders extrapolating of these findings from non-ICU ward patients to critically ill patients uncertain.

Inflammatory biomarkers propose a promising approach for assessing severity of the inflammatory response, organ function and disease progression in greater detail [38–40]. Understanding the intricate relationship between drug exposure and changes in biomarker dynamics is critical for advancing precision dosing strategies in critically ill populations [41].A deeper insight into this relationship is crucial, as it holds the potential to refine precision dosing strategies in critically ill patients thereby improving patient outcomes.

Thus, this review endeavors to synthesize the existing evidence regarding the relation between inflammatory biomarkers and pharmacokinetics in critically ill patients. The purposes of this study are to reveal how inflammation changes drug behavior in this vulnerable patient group, which may provide inspiration for future pharmacokinetic clinical trials in critically ill patients that ultimately improve patient outcomes in the future.

## Methods

### Search strategy

This review was conducted in line with the PRISMA (Preferred Reporting Items for Systematic Reviews and Meta-Analyses) guidelines. We performed a comprehensive search of databases including Medline, EMBASE, Web of Science Core Collection, and the Cochrane Library to gather all relevant articles discussing the influence of inflammatory biomarkers on the PK of critically ill patients. The search combined terms for (1) pharmacokinetics or pharmacodynamics, (2) inflammation or inflammatory markers such as TNF-alpha or interleukins, and (3) intensive care or critical illness. The search, limited to articles published in English, was completed by November 2023 and carried out independently by two researchers (LL and JZ). All selected articles were independently reviewed by LL and JZ. Disagreements were resolved by a third senior reviewer (SS). The specific search terms employed are detailed in Supplementary File 1.

### Eligibility criteria

The initial screening involved examining the titles and abstracts of the retrieved studies by two independent researchers, with the exclusion of irrelevant articles. The inclusion criteria were as follows: original, peer-reviewed articles focusing on PK research in ICU settings, which incorporated at least one inflammatory biomarker over time as outlined in our search strategy (Supplementary File 1). Titles and abstracts were scrutinized to identify pertinent studies. Exclusion criteria included articles not pertaining to ICU settings, review papers, case studies and studies without biomarkers. Additionally, reference lists of selected studies were reviewed to identify further relevant articles. All references from these searches were managed using Endnote X9^®^.

### Data extraction

For each included study, we extracted and collated key data comprising the author(s), year of publication, study drugs involved, number of participants, and specific effects of the biomarker(s) on the drug’s absorption, distribution, metabolism, and excretion (ADME).

## Results

**Fig. 2:**
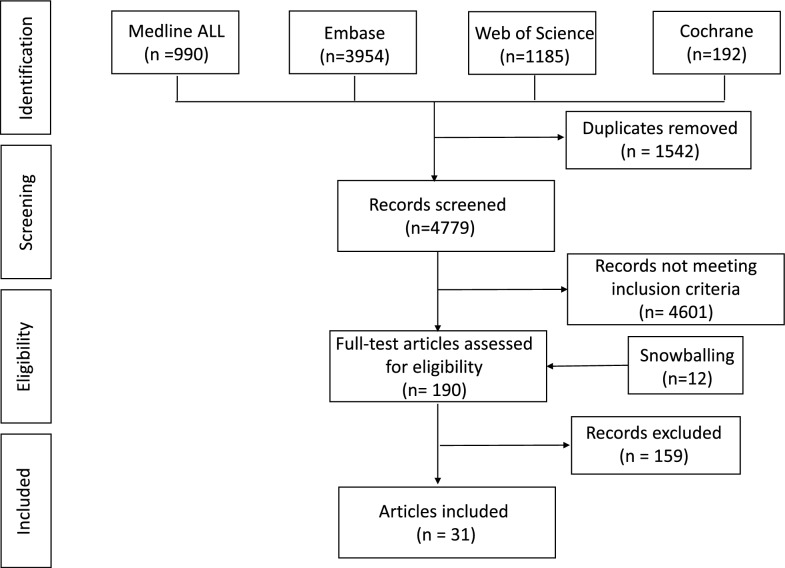
PRISMA flow diagram. Snowballing is a method of tracking down relevant publications using the bibliography or reference list at the end of an article as a starting point

The process of selecting studies for our review is comprehensively outlined in Supplementary File 1. Initially, a search of the relevant literature on the impact of inflammation on pharmacokinetics in the critically ill yielded 6,321 articles. After removing duplicates using the “Bramer method” in EndNote [42], we screened the titles and abstracts of 4,479 articles. This led to the identification of 190 studies that fulfilled our eligibility criteria and were subjected to a full-text review. Ultimately, 31 papers were included in our analysis, based on inclusion and exclusion criteria, as illustrated in Fig. 2.

### Absorption

The current research exploring the impact of inflammation on drug absorption is scarce. However, our review identified two drugs, prasugrel [43] and tobramycin [44], that exhibit a correlation between their systemic exposure and CRP levels, albeit in different ways (Table 1). The active metabolite of prasugrel was inversely correlated with CRP levels, which may indicate a reduced absorption of the metabolite into the systemic circulation. This finding is consistent with another antiplatelet medication, clopidogrel, whose bioavailability was lower in critically ill patients [45]. In contrast, tobramycin which administered enteral 6 mg of four times daily, used for selective digestive tract decontamination, showed an opposite trend. Increased CRP levels were associated with increased tobramycin levels (maximum concentration range from 0.27 to 1.3 mg/L) in the blood, which may indicate tobramycin leakage from the gut related to shock due to extensive inflammation [44]. This finding is particularly significant, considering another study reported instances of acute renal failure potentially linked to tobramycin intoxication during the decontamination process [46]. No studies reported on the effect of inflammation on drug absorption subcutaneously or by other routes. These studies did not explore the relationship between inflammatory biomarkers and drug Cmax and tmax, and therefore could not reflect the effect of inflammation on drug absorption rate. These varied correlations highlight the complex and drug-specific nature of how inflammation can influence drug absorption and systemic exposure.

**Table 1:** Inflammation effect on drug absorption*

Type	Drug	Time	Patient	Number	Biomarker	Range	Parameter	Relationship of biomarker and parameter	influence on concentration
Antiplatelet agents	Prasugrel [43]	2017	Adults	23	CRP	128 (IQR 89–164) (mg/l)	Prasugrel active metabolites concentration	Negative	inflammation will lower prasugrel active metabolite
Antibiotic	Tobramycin [44]	2011	Adults	100	CRP	26.0 (IQR 3.0 to 160.6) (mg/l)	Tobramycin concentration	Positive	Inflammation will increase the systematic tobramycin exposure

Table shows medians and ranges(), unless specified otherwise

*IQR* interquartile range

*More detailed contents in table can be found in the supplementary excel file

So research on absorption is scarce and at the same time there are numerous parameters in critically ill patients who will have an effect on absorption which are related to inflammation [47–50]. Many critically ill patients suffer of impaired motility of the digestive tract, including gastroparesis, which might result in limited passage of drugs from the stomach to the lower digestive tract [51, 52]. The function of the latter is also impaired many times: transit time is decreased, or increased, and permeability can be affected by impaired mesenteric blood flow and intestinal edema [53]. Microbiome will be altered due to the cause of disease, medication, of critical illness itself, resulting in altered enteral metabolism [54]. Next to this, pH of the stomach will be increased, etc.

Take home messages for drug absorption: in general, though literature is limited, one should take into account that there are numerous processes in the inflamed critically ill patients that hamper enteral absorption, in particular: (1) For oral drugs that require hepatic metabolism for activation, dose adjustments may be necessary in patients with high levels of inflammation. (2) Be cautious when administering drugs intended for local gastrointestinal action (e.g., selective digestive decontamination) to patients with elevated inflammation. (3) Whenever feasible, consider TDM for oral drugs in critically ill patients to ensure optimal exposure and mitigate potential adverse events due to the high variability in their PK.

### Distribution

Table 2 delineates the impact of inflammation on the distribution of various drugs, including those processed via hepatic and renal pathways [55–62]. A notable finding is the positive correlation between inflammatory markers, such as WBC [60] and ESR [56], and the drugs’ volume of distribution (Vd). This correlation suggests that escalating levels of inflammation may lead to glycocalyx injury and endothelial disruption, resulting in increased extracellular body water and enhanced fluid translocation. Consequently, to achieve a goal concentration, an increase in the initial dose may be required. Specifically, in a drug with high protein binding, such as temocillin, CRP demonstrates a significantly negative correlation with the binding capacity of plasma proteins, resulting in an increased unbound fraction of the drug [57]. Furthermore, our analysis shows that inflammation might facilitate the penetration of certain drugs, like meropenem [58], and morphine metabolites [61, 62] into the cerebrospinal fluid (CSF). Conversely, amphotericin B presents a contrasting scenario [59]. Its peritoneal concentration is inversely related to CRP levels, indicating a decrease in its ability to penetrate the peritoneal cavity as inflammation levels rise.

**Table 2:** Inflammation effect on drug distribution*

Type	Drug	Time	Patient	Number	Biomarker	Range	Parameter	Relationship of biomarker and parameter	influence on concentration
Antibiotic	Chloramphenicol [60]	2000	Children	30	WBC	9.79 ± 3.71 (SD) (10^3^/mm^3^)	Vd/F	Positive	high WBC may lower the Cmax
Antibiotic	Amikacin [56]	2022	Adult	21	ESR	53 (IQR 33.1–77.3) (mm/hour)	Vd	Positive	high WBC may lower the Cmax
Antibiotic	Temocillin [57]	2022	Adult	37	CRP	ICU group: 179 (range 50.9–550) (mg/L)	Protein binding	Negative	High CRP will have higher free unbound concentration
Antifungal	Amphotericin B [59]	2007	Adult	21	CRP	NA	Peritoneal concentration	Negative	High CRP will have lower peritoneal concentration
Antibiotic	Meropenem [58]	2022	Adult	51	IL-6	4855 (IQR 273–5000) (pg/mL)	CSF concentration	Positive	high IL-6 will have high CSF concentration
Analgesics	Morphine [61]	2017	Adult	16	IL-6	Plasma: open vs endovascular surgery 105 (IQR 40–256); 29 (IQR 16–70) (pg/mL) CSF: open vs endovascular surgery 79 (IQR 26–133); 16 (IQR 9–80) (pg/mL)	CSF morphine metabolites concentration	Positive	high IL-6 will have higher morphine metabolites conentration
Analgesics	Morphine [62]	2009	Adult	33	IL-6	NA	CSF morphine metabolites concentration	Positive	CSF IL-6 correlate with higher morphine metabolites

Table shows medians and ranges(), unless specified otherwise

*IQR* interquartile range, *NA* not available, *Vd* volume of distribution, *WBC* white blood cell, *ESR* erythrocyte sedimentation rate, *CSF* cerebrospinal fluid

*More detailed contents in table can be found in the supplementary excel file

Critically ill patients often experience significant fluid shifts into interstitial spaces, which can significantly alter drug exposure by increasing their volume of distribution, especially for those hydrophilic drugs like aminoglycosides and beta-lactams [63–82]. However, few studies have quantitatively explored the relationship between inflammatory biomarkers and peak concentrations. In addition, studies have shown that drug concentrations at the target site may not be consistently reflected in blood levels [15, 83–85]. Inflammation may significantly influence the distribution and penetration processes, potentially resulting in altered peak and target drug concentrations.

Numerous physiological factors can influence drug distribution in critically ill patients. These include fluid sequestration, weight changes, albumin levels and neurological pathology, all of which can be significantly impacted by systemic inflammation. The systemic inflammatory response syndrome (SIRS) has been associated with increased fluid sequestration [86, 87], resulting fluid accumulation and impaired fluid removal can significantly alter the volume of distribution for many drugs. Moreover, body weight changes in critically ill patients, often attributed to fluid balance [88], can further complicate drug dosing. Furthermore, hypoalbuminemia is common in critically ill patients, and high levels of inflammation are often associated with low albumin levels [89, 90], which may significantly affect the concentrations of highly protein-bound drugs. In addition, neurological pathology and alterations in the blood–brain barrier (BBB) during systemic inflammation can impact drug penetration, leading to endothelial cell damage and associated with cerebral edema, which lead to altered drug distribution [91–94]. Lastly, inflammation is associated with glycocalyx degradation which could lead to microcirculatory dysfunction, thus influencing the target drug concentration [95]. These complex interactions between inflammation and various physiological factors underscore the challenges of achieving optimal drug dosing in critically ill patients.

Inflammation influence drug distribution through multifaceted mechanisms affecting both drug-binding proteins and transporters. The acute-phase response elevates AAG levels [96], potentially altering the free fraction of drugs bound to it, impacting their distribution and efficacy. Concurrently, inflammation affects the blood–brain barrier by downregulating efflux transporters such as P-gp, BCRP, and MRP2 [97], promoting drug penetration into the CNS and potentially influencing drug pharmacokinetics and pharmacodynamics in inflammatory conditions. However, it is crucial to note that research specifically investigating these effects in the ICU setting remains limited. These complex relationships between inflammation and drug distribution emphasize the need for more relevant studies in future.

Take home messages for drug distribution: In general, distributive volume is highly variable in the critically ill patients with high levels of inflammatory biomarkers. Distribution volume can be higher due to volume resuscitation, lower due to excessive fluid loss, protein binding is altered due to altered albumin levels, interaction, etc. and permeability of vessels, but also barriers, including the blood–brain barrier will be changed. In particular: (1) For patients with more severe inflammation, highly hydrophilic drugs may require higher loading doses. (2) Be careful for high protein binding drugs, which inflammation might cause large free drug concentration change. 3. Inflammation might change the drug penetration ability, but may have different effect in different sites.

### Metabolism

Our findings show that inflammation affects metabolism of a diverse array of drugs, as shown in Table 3. The general trend in the relationship between inflammation and metabolism is that increased levels of inflammation are associated with decreased drug clearance, resulting in increased drug exposure, with the exception of two drugs, chloramphenicol and tramadol. This phenomenon is not limited to drugs metabolized by phase-I enzymes like Cytochrome P450 enzymes (CYP) but also extends to those primarily processed by phase-II enzymes like UDP-glucuronosyltransferase (UGT), including chloramphenicol [60] and haloperidol [98]. In the case of chloramphenicol, although there is a positive correlation between WBC counts and creatinine clearance, the augmentation in the Vd is more substantial compared to the increase in CL. This suggests that, overall, there is a decrease in the drug’s elimination rate. A notable exception is found with tramadol, metabolized by CYP2B6, where its metabolite levels are elevated during inflammation, as indicated by lower cholinesterase activity [99]. This observation aligns with the findings of Camille Lenoir et al., who reported increased CYP2B6 enzyme activity during acute inflammation [100].

**Table 3:** Inflammation effect on drug metabolism*

Type	Drug	Time	Patient	Number	Biomarker	Range	Parameter	Relationship of biomarker and parameter	influence on concentration
Antibiotic	Chloramphenicol [60]	2000	Children	30	WBC	9.79 ± 3.71 (SD) (10^3^/mm^3^)	CL	Positive	High WBC will may have lower drug exposure
Corticosteroid	Dexamethasone [117]	2023	Adult	18	CRP	20 (range 0.6–449) (mg/L)	CL	Negative	High CRP will have higher dexamethasone concentration
Antipsychotic	Haloperidol [98]	2022	Adult	22	CRP	171 (range 4.1–368) (mg/L)	CL	Negative	High CRP will have higher haloperidol concentration
Antineoplastic agent	Imatinib [55]	2021	Adult	134	AAG and CRP	AAG: 1.96 (IQR 1.6–2.3) (g/L) CRP: 110 (IQR 63–171) (mg/L)	CL	Positive	High AAG have high Imatinib AUC; High CRP have high Imatinib AUC
Antiretroviral	Lopinavir [118]	2020	Adult	92	CRP	65 (IQR 36–113) (mg/L)	Plasma concentrations	Positive	High CRP will have higher lopinavir concentration
Benzodiazepine	Midazolam [119]	2022	Adult	38	IL-6	109 (range 2–5987) (pg/mL)	CL	Negative	High IL-6 will have higher midazolam concentration
Benzodiazepine	Midazolam [120]	2016	Neonate and children	83	CRP	32 (range 0.3–385) (mg/L)	CL	Negative	High CRP will have higher midazolam concentration
Benzodiazepine	Midazolam [121]	2022	Adult	48	CRP	113.6 (mg/L)	α-hydroxymidazolam/midazolam plasma ratio	Negative	High CRP will have lower midazolam metabolite concentration and CYP3A activity
Benzodiazepine	Midazolam [122]	2018	Children and adult	136	CRP	NA	CL	Negative	High CRP will have higher midazolam concentration
Benzodiazepine	Midazolam [123]	2022	Children	54	CRP	37.5 (range 5–306) (mg/L)	CL	Negative	High CRP will have higher midazolam concentration
Barbiturate	Pentobarbital [124]	2023	Neonates and children	45	CRP	Validation group: 193 (IQR 75–295) (mg/L)	CL	Negative	High CRP will indicate high Pentobarbital concentration
Analgesic	Tramadol [99]	2021	Adult	47	ChE	Normal 6,171 (IQR 4777–6635); Low: 3230 (IQR 2837.5–2766.5) (U L^−1^)	N-demethyltramadol AUC	Negative	systemic inflammation associated with higher tramadol metabolites
Antifungal	Voriconazole [125]	2019	Adult	33	CRP	NA	Plasma concentration	Positive	CRP value > 100 mg/L associated with voriconazole concentrations > 5.5 mg/dL
Antifungal	Voriconazole [126]	2016	Adult	7	CRP	NA	Plasma concentration	Positive	Higher CRP were associated with higher trough concentration
Antifungal	Voriconazole [127]	2023	Adult	50	CRP and PCT	CRP: 73.6 (IQR 35.2–166.1) (mg/dL) PCT: 0.38 (IQR 0.10–1.03) (ng/mL)	Voriconazole metabolites	Negative	CRP value > 114.6 mg/L was associated with Cmin > 3 mg/L; PCT value > 0.3 ng/mL was associated with Cmin > 3 mg/L
Antifungal	Voriconazole [128]	2023	Adult	9	CRP	103.79 ± 83.72 (SD) (mg/L)	Plasma concentration	Positive	Higher CRP were associated with higher trough concentration
Antifungal	Voriconazole [129]	2023	Adult	15	CRP	91 (range 0.3–434) (mg/L)	CL	Negative	Higher CRP were associated with higher trough concentration
Anticonvulsant	Phenytoin [130]	1997	Adult	9	IL-6	11.4 ± 186.0 pg/ml	maximum velocity (Vmax) of phenytoin metabolism	Negative	High levels of IL-6 associated with high phenytoin concentration

Table shows medians and ranges(), unless specified otherwise

*IQR* interquartile range, *NA* not available, *WBC* white blood cell, *CL* clearance, *AAG* alpha-1-acid glycoprotein, *ChE* cholinesterase activity, *AUC* area under the plasma drug concentration–time curve

^*^More detailed contents in table can be found in the supplementary excel file

For drugs that eliminated by liver, inflammation may influence these drug in two primary ways: by altering hepatic blood flow, which impacts drugs with high extraction rates (e.g., morphine, propofol) [101], or by modifying hepatic enzyme activity, affecting drugs with low extraction rates (e.g., midazolam, ceftriaxone) [102, 103].

Most current research focusing on the impact of inflammation on drug pharmacokinetics has been centered on the metabolism phase [104] [105–109]. Consistent with this, inflammatory biomarkers such as IL-6, TNF-α, and CRP negatively correlate with CYP drug metabolizing capacity [16, 17, 105, 110–113]. In contrast, increased hepatic blood flow in critically ill patients, combined with minimally affected phase-II metabolism, can lead to unchanged or even enhanced drug clearance [114–116]. Our review showed that in critically ill patients with high levels of inflammatory biomarkers often exhibit reduced clearance of drugs metabolized by the liver, raising the risk of drug overexposure [113]. Key treatment areas in the ICU, such as anti-inflammatory, sedative, analgesic, anticoagulant, antiarrhythmic, and anti-infection therapies, largely depend on liver metabolism and have narrow therapeutic windows. These findings collectively underscore the multifaceted ways inflammation can impact drug metabolism, significantly influencing drug concentrations in critically ill patients.

Take home messages on metabolism: In general the clinician should be aware that metabolism is altered due to inflammation [16] and dose adjustments possibly necessary but highly complex. In particular For drugs undergo hepatic elimination, lower maintenance doses may be needed during periods of high inflammation. Once inflammation resolves, these adjustments should be reversed to maintain optimal efficacy. For high first pass drugs, they should dose lower, since inflammation will inhibit drug metabolism. However, if the drug has active metabolites, should dose higher.

### Excretion

Drugs and their metabolites are eliminated via multiple excretory routes, such as renal (urine), hepatic (bile), and through excretion in sweat, saliva, gastrointestinal secretions, expired air from the lungs, tears, and breast milk. In critically ill patients, we found that the presence of inflammation greatly affected on drug excretion through the kidneys, as shown in Table 4. For instance, beta-lactam antibiotics such as meropenem and piperacillin show higher drug concentrations in the presence of elevated levels of inflammatory biomarkers. However, the anticoagulant nadroparin exhibits decreased exposure under conditions of increased inflammation. Similarly, it is observed with vancomycin in neonates, where high inflammation correlates with increased drug clearance and consequently lower drug concentrations which might be due to fluid overload, capillary leak, and renal hyper filtration.

**Table 4:** Inflammation effect on drug excretion*

Type	Drug	Time	Patient	Number	Biomarker	Range	Parameter	Relationship of biomarker and parameter	influence on concentration
Antifungal	Flucytosine [149]	2000	Adult	53	Thrombocyte nadir	NA	CL	Positive	Thrombocyte nadir are negatively correlated with drug exposure
Antibiotic	Linezolid [150]	2016	Adult	52	Fibrinogen	(Range 2.6–24.4) (μmol/liter)	CL	Positive	decrease fibrinogen is associated with linezolid concentration
Antibiotic	Meropenem [139]	2022	Adult	12	CRP	106.5 (12–383) (mg/L)	CL	Negative	high CRP will indicate high meropenem concentration
Anticoagulants	Nadroparin [151]	2022	Adult	65	CRP, D-dimers	CRP 61.0 (IQR 32.5–101) (mg/L) D‐dimer 1.24 (IQR 0.85–2.18) (mg/L)	CL	Positive	CRP and D-dimers will lead to decrease nadroparin; Corticosteroids will lead to increase nadroparin exposure
Antibiotic	Piperacillin [152]	2021	Adult	160	CRP and IL-6	CRP (range 11–605) (mg/L) IL-6 (range 4–35,500) (pg/mL)	Plasma concentration	Positive	High CRP will indicate toxic concentration while low might indicate subtherapeutic conentration; IL-6 low might indicate subtherapeutic concentration
Antibiotic	Vancomycin [153]	2022	Neonatal	52	CRP	13.5 (IQR 5–41.5) (mg/L)	Plasma concentration	Negative	systemic inflammation will lead to low vancomycin concentration

Table shows medians and ranges(), unless specified otherwise

*IQR* interquartile range, *CL* clearance, *NA* not available

^*^More detailed contents in table can be found in the supplementary excel file

For drugs eliminated renally, research on the impact of inflammation on renal drug excretion remains limited. Critically ill patients experience complex pathophysiological changes that can either enhance or impair renal function [14, 68, 115, 131–135]. Our review primarily identified a negative correlation between inflammation and drug clearance, potentially due to the association between high inflammatory status and kidney injury [15, 136–138]. Inflammatory markers such as CRP, IL-6 and IL-18 have been linked to organ failure and could be indicators of acute kidney injury [19, 139, 140]. In one study on end-stage renal disease patients, biomarkers including vWf, sVCAM‐1 were found to be correlated with renal functions [141]. In addition, one study found in critically ill patients, inflammation may lower the urine output [19]. Conversely, some studies suggest that inflammation can elevate cardiac output and enhance glomerular filtration rate, potentially leading to increased drug clearance [18, 19, 142, 143], a phenomenon often observed in clinical practice as augmented renal clearance [133]. This could also explain the exception noted in our review that the clearance of nadroparin and vancomycin increased with increasing inflammation.

In critically ill patients, drug excretion is influenced by various factors, including augmented renal clearance (ARC), renal replacement therapies (RRT), and fluid overload. Inflammation may be a key factor underlying many of these changes. Inflammation can trigger ARC through mechanisms like vasodilation of afferent glomerular arterioles and increased cardiac output leading to increased renal blood flow [133, 144]. While CRRT can directly clear certain drugs, it may also remove inflammatory cytokines and promote the recovery of acute kidney injury (AKI), thus indirectly regulating drug excretion [145]. Furthermore, fluid overload, often associated with inflammation-induced endothelial dysfunction and subsequent renal injury [146], can significantly impair renal recovery [147] and reduce drug clearance [148]. Thus, volume status, renal function, and the use of renal replacement therapies all interact with the inflammatory state to affect drug excretion in critically ill patients.

Take home messages for drug excretion: The influence of inflammation on the renally excreted drugs is complex and varies depending on the specific drug and patient population (e.g., adults vs. pediatrics). Clinicians should closely monitor renal function and drug concentrations in patients with inflammation to ensure optimal dosing and minimize the risk of adverse events.

### The impact of inflammation on ADME and clinical considerations

**Fig. 3:**
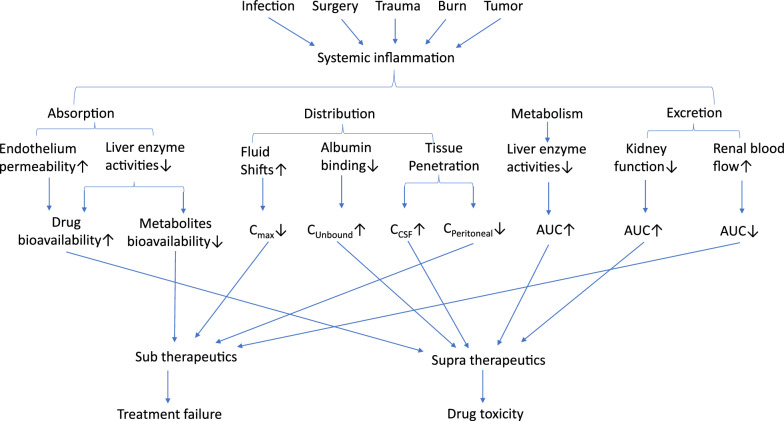
summary of inflammatory biomarkers on PK in critically ill patients. AUC: Area Under the Curve; C_max_: Maximum concentration; C_unbound_: Free drug concentration; C_CSF_: Cerebrospinal fluid concentration; C_Peritoneal_: Peritoneal drug concentration

Inflammation is a hallmark of critically ill disease and its level is measured in daily practice by different biomarkers. Our review summarizes the complex relationship between inflammatory biomarkers and PK parameters in critically ill patients. To unravel this complex relation we deduced it to the 4 different components of pharmacokinetics: absorption, distribution, metabolism and elimination. While previous research has suggested that inflammation can influence drug behavior in patients, most of these studies have primarily focused on specific CYP metabolism enzymes [154, 155], on patients in non-ICU wards[37, 156, 157] or only on certain aspects of the process, such as drug penetration [83, 84] or metabolism [103]. The impact of inflammation on PK (i.e., downregulation of different enzymes and transporters) is well known, but the relationship with drug exposure in critically ill patients has not been fully appreciated. Our review highlights the multifaceted interplay between inflammation and pharmacokinetics (PK), often underestimated in previous pharmacokinetic studies. These complex interactions can lead to unpredictable drug exposures, particularly in critically ill patients under different inflammatory states (Fig. 3).

### Complex relationship between inflammation and PK

**Fig. 4:**
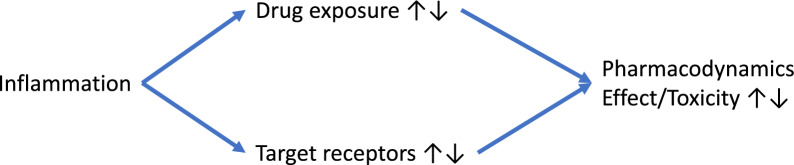
Inflammation effect on pharmacodynamics

The relationship between the inflammation and drug exposure is complex. In this review, we observed that high levels of inflammation are generally associated with increased drug exposure in critically ill patients. However, findings are not consistent across all drugs and also differ across different patient groups, as our review found in the certain drugs like echinocandins, new triazoles (posaconazole and isavuconazole) and hydroxychloroquine (Table 5) did not show correlations between inflammatory biomarkers and drug exposure. One reason why the pharmacokinetics of certain drugs are not significantly influenced by inflammation may be that studies often assess only one inflammatory biomarker, such as CRP. Additionally, many of these medications, including echinocandins (e.g., anidulafungin, caspofungin) and newer triazoles (e.g., posaconazole, isavuconazole), are not primarily metabolized by CYP enzymes, making them less susceptible to changes caused by inflammation. In addition, for non-critically ill patients, there are differences in the impact of inflammation on PK (Supplementary Table 2). These discrepancies highlight the diverse and complex effects of inflammation, influenced by patient condition and specific treatment. The effects of inflammation on some drugs were compared between ICU and non-ICU settings (Supplementary Table 3). Overall, the impact of inflammation on pharmacokinetics appears to be consistent across both ICU and non-ICU setting. Take home messages for more general ICU drugs: Even in the non-ICU setting, inflammation can affect PK, and ICU physicians should be aware that in critically ill patients, inflammation is more severe and may have a more profound effect on PK. This makes extrapolation from non-ICU to ICU more difficult (Fig. 4).

**Table 5:** drugs used in critically ill patients that are not influenced by the inflammation

Drug Class	Elimination pathway	Drug	Time	Author	pt group	number	Effect
Antibacterial	Renal	Amikacin	2014	C. M. T. Sherwin	Children	70	CRP and platelet count were not associated with CL
Antibacterial	Renal	Amikacin	2010	I. K. Delattre	Adults	88	CRP has no relationship with CL
Anti-fungal	slow chemical hydrolysi	Anidulafungin	2013	P. Liu	Adult	21	PK did not differ between healthy and ICU patients
Anti-fungal	slow chemical hydrolysi	Caspofungin	2020	Agnieszka Borsuk-De Moor	Adult	30	No covariate (PCT) could explain the variability
Antibacterial	Renal	Ceftriaxone	2022	Sonya Tang Girdwood	Children	195	CRP did not affect the CL of total and free ceftriaxone
DMARDs	Hepatic	Hydroxychloroquine	2023	A. K. Lyashchenko	Adult	421	Variability in systemic exposure could not be clearly explained by inflammatory status
DMARDs	Hepatic	Hydroxychloroquine	2020	Catia Marzolini	Adult	51	No correlation between hydroxychloroquine concentrations and CRP
Analgesics	Hepatic	Ketobemidone	2002	A. Al-Shurbaj	Adult	17	No relationship between CRP/WBC and CL
Anti-fungal	Hepatic	Isavuconazole	2022	Léa Bolcato	Adult	33	No relationship between CRP and CL
Antibacterial	Renal	Linezolid	2017	I. K. Minichmayr	Adult	51	No relationship between CRP and PK parameters
Anti-fungal	Hepatic	Posaconazole	2019	Anne-Grete Märtson	Adult	55	No relationship between inflammatory biomarkers and CL
Anesthetic	Hepatic	Propofol	2016	P. Smuszkiewicz	Adult	29	No relationship between CRP and CL

### Inflammation and pharmacodynamics

The effects of inflammation on pharmacodynamics are multifaceted (Fig. 4). On the one hand, inflammation can indirectly influence drug efficacy and safety by altering pharmacokinetics, leading to supra- or sub-therapeutic drug concentrations. On the other hand, inflammation can downregulate certain drug receptors, such as calcium channel blockers, β-adrenoreceptors, and potassium channel antagonists, reducing their efficacy despite increased plasma drug concentrations [156, 158]. Furthermore, the interplay between inflammation’s direct and indirect effects on PD can further complicate the clinical picture.

For the PK-related influence on PD, piperacillin and voriconazole demonstrate how high inflammation can lead to elevated drug concentrations and increased toxicity [125, 152]. For the inflammation effect on PD, in midazolam, increased drug exposure occurs during inflammation; however, reducing the dosage is not recommended [159]. And in the case of prasugrel, the reduced antiplatelet effect may be attributed not only to decreased levels of its active metabolite but also to inflammation-induced increases in platelet reactivity.

Despite some evidence indicating that inflammation can lead to toxicity in both ICU and non-ICU settings, research exploring the direct relationships between inflammatory biomarkers and PD outcomes remains limited. Now sometimes this is not a problem in daily practice, as the pharmacodynamic effect can be closely monitored, for example vasopressors or sedatives, or easily measured, for example heparin and vancomycin, but for other drugs this is not, including certain antibiotics, immunomodulators and antiplatelets, both essential drugs for the disease they are prescribed for.

Take-home message: Clinicians should carefully consider dosing adjustments for patients with high inflammatory states, balancing the potential risks and benefits of medication administration under these complex conditions.

### Promising biomarkers

**Fig. 5:**
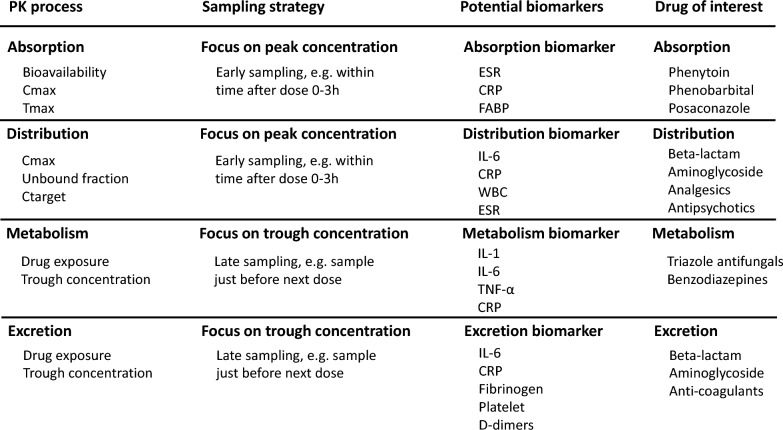
Suggestions for future ICU pharmacokinetic clinical trials. ESR: Erythrocyte Sedimentation Rate; FABP: Fatty acid-binding protein; WBC: white blood cell. Cmax: Maximum concentration; Tmax: Time to maximum concentration; Ctarget: Target concentration; ESR: Erythrocyte Sedimentation Rate; CRP: C-Reactive Protein; IL-1: Interleukin-1; IL-6: Interleukin-6; WBC: White Blood Cell Count; TNF-α: Tumor Necrosis Factor-alpha

Inflammatory biomarkers hold promise for guiding drug dosing in critically ill patients. In our review, CRP appears to be the biomarker most consistently associated with alterations in pharmacokinetic parameters, particularly those related to metabolism. This observation may be attributable to the prevalent monitoring of CRP as an inflammatory biomarker in critically ill patients compared with other inflammatory biomarkers (IL-6, IL-8, etc.). At the same time, CRP is a rather non-specific inflammatory biomarkers There are other promising inflammatory, including more specific, biomarkers, of interest (Supplementary file 4). An ideal biomarker should be both specific and easily available. Unfortunately, we need more research for this, and CRP will until then by the best surrogate. Based on the results of our review, we propose a targeted approach to refine future pharmacokinetic clinical trial designs (shown in Fig. 5). By tailoring trial protocols to specific therapeutic agents (e.g., anticoagulants, anti-infectives, and analgesics), we aim to determine the most relevant pharmacokinetic parameters (such as peak concentration, free drug concentration, target organ tissue concentration, steady-state concentration, etc.). Incorporating more specific inflammatory biomarkers will better elucidate PK variability among critically ill patients, potentially leading to improved clinical outcomes. And in future PK studies in critically ill patients, longer follow-up period is needed since the inflammation effects on PK parameters may differs in different patterns of inflammation (pro- or anti-inflammatory, early or late) which already found in certain drugs like vancomycin [160, 161]. AI could be a valuable tool for precision dosing, given its ability to manage large datasets and enhance biomarker identification [162]. This is especially beneficial for critically ill patients experiencing dynamic inflammatory conditions and requiring continuous monitoring. Take home messages for promising biomarkers: Inflammatory biomarkers that reflect organ functions (especially for liver and renal functions) can be invaluable in improving precision dosing in critically ill patients, ensuring both efficacy and safety in treatment protocols.

### Limitations

Our review has several limitations. The main limitation is that due to limited literature and evidence, this review is limited to pharmacokinetics and does not consider pharmacodynamics. Next to this, we had to reduce real-life complexity by using a classic pharmacokinetic approach (ADME), but effects on pharmacokinetics will never be limited to only one of these parameters. Third, the number of drugs found in literature is limited, especially regarding all 4 pharmacokinetic parameters. Fourth, the search was restricted to English-language publications, potentially excluding relevant research in other languages. Fifth, by focusing on studies incorporating inflammatory biomarkers, we may have missed studies that qualitatively explored the impact of inflammation on PK, though they lacked quantitative measures. Sixth, the evidence linking inflammatory markers to PK parameters in critically ill patients remains sparse and inconsistent, limiting the generalizability of our findings. Seventh, attributing PK changes solely to inflammation in the complex ICU environment is challenging due to confounding factors such as fluid therapy, CRRT, disease severity, and renal function, which often correlate with inflammation. Eighth, data on drug interactions and comedication, crucial in the polypharmacy setting of most ICUs, were often missing from included studies. Ninth, while the impact of inflammation on drug pharmacokinetics may exhibit similarities in non-ICU settings, direct extrapolation between these settings should be avoided due to the significant differences in the severity and nature of inflammation. Finally, the lack of studies investigating the relationship between inflammation and pharmacodynamics in critically ill patients hinders our ability to provide precise dosing recommendations for clinicians. Future research should address these limitations to improve our understanding of the complex interplay between inflammation and drug disposition in critically ill patients.

## Conclusion

Our review underscores the importance of inflammation as a key factor influencing the complex drug PK behavior in the critically ill patients. To optimize treatment efficacy, one should consider each patient’s specific inflammatory profile. Furthermore, future PK-related clinical trials conducted in critically ill patients should consider the heterogeneity of patients’ inflammatory status. This involves monitoring inflammatory biomarkers in critically ill patients, which will provide a clearer understanding of the interplay between inflammation, organ function, and PK behavior across diverse patient groups.

## Supplementary Information

Additional file 1.

Additional file 2.

Additional file 3.

## Data Availability

No datasets were generated or analysed during the current study.

## Abbreviations

AAG: Alpha-1-acid glycoprotein
ADME: Absorption, distribution, metabolism, and excretion
AKI: Acute kidney injury
ARC: Augmented renal clearance
ARDS: Acute respiratory distress syndrome
AUC: Area under the curve
BBB: Blood–brain barrier
BCRP: Breast cancer resistance protein
CL: Clearance
Cmax: Maximum concentration
CRP: C-reactive protein
CSF: Cerebrospinal fluid
Ctrough: Trough concentration
CYP: Cytochrome P450
EMBASE: Excerpta medica database
ESR: Erythrocyte sedimentation rate
ICU: Intensive care unit
IL-6: Interleukin-6
MIC: Minimum inhibitory concentration
MODS: Multiple organ dysfunction syndrome
MRP2: Multidrug resistance-associated protein 2
PCT: Procalcitonin
PD: Pharmacodynamics
P-gp: P-glycoprotein
PK: Pharmacokinetics
PRISMA: Preferred reporting items for systematic reviews and meta-analyses
RRT: Renal replacement therapy
SIRS: Systemic inflammatory response syndrome
TNF-α: Tumor necrosis factor-alpha
tmax: Time to reach peak concentration
UGT: UDP-glucuronosyltransferase
Vd: Volume of distribution
WBC: White blood cell
