# Supplementary material for: The relation between inflammatory biomarkers and drug pharmacokinetics in the critically ill patients: a scoping review
Source: Crit Care. 2024 Nov 19;28:376. doi: 10.1186/s13054-024-05150-4 (PMC11577668; doi:10.1186/s13054-024-05150-4)
Supplement: Supplementary file 1 — Additional file 1. [file 13054_2024_5150_MOESM1_ESM.docx]

Supplementary table 1 Summary of inflammatory biomarkers included in our review.

| Marker | Type | Produced by | Time to peak | Duration (half-life) | Reference range |
| --- | --- | --- | --- | --- | --- |
| ESR[1] | Cell | Bone marrow | 24–48h | 5 days | ≤ 15-20 mm/hr |
| WBC[2, 3] | Cell | Bone marrow | 2-4 hours | 7-10 days (1-2day) | 4000-10,000 cells/microliter |
| Thrombocyte[2, 4] | Cell fragments | megakaryocytes | 2 weeks | 2 days | 150 to 400 × 109/L |
| CRP [1, 5-7] | Protein | Liver | (6-24h) | 7-10 days (19h) | 0-10 mg/L |
| PCT [1, 6-8] | Protein | Thyroid gland | 6-24 hours | 1-3 days (20-36h) | 0.1-0.5 ng/mL |
| Cholinesterase[9, 10] | Protein | Liver | 1h | (2-3 days) | 5,000–12,000 U/L |
| Fibrinogen[5, 7] | protein | Liver | 12h | NA | 200 and 400 mg/dL |
| D-Dimer [6, 11] | Protein | fibrin | 3-7 days | (8h) | <0.5 |
| alpha1‐acid glycoprotein (AAG) [7, 12] | protein | liver | 12-24h | 7 days | 0.1 to 0.32 mg/m |
| IL-6 [6, 13, 14] | Cytokine | Various cells | Within 1 day | 1-3 days | 2-8 pg/mL |

Supplementary table 2 inconsistency of inflammatory effect on drug PK in general ward patients

| Drug | Time | author | pt group | pt number | Effect |
| --- | --- | --- | --- | --- | --- |
| Carboplatin | 2020 | Benjamin D W Harris | NSCLC | 61 | Neutrophil–lymphocyte ratio is inversely correlated with CL |
| Doxorubicin | 2016 | Jonás Samuel Pérez-Blanco | Cancer | 45 | neutropenia grade (P = 0.068) and the neutrophil counts (P = 0.089) showed negative correlation to drug AUC |
| Clofarabine | 2004 | Peter L Bonate | acute leukemia | 40 | WBC is positive correlated with V1 |
| Etoposide | 2012 | Mikkel Krogh-Madsen | acute myeloid leukemia | 23 | WBC is positive correlated with CL |
| Midazolam | 2017 | Linda G Franken | terminally ill adult patients | 45 | CRP is negative correlated with CL |
| Voriconazole | 2022 | Takuto Takahashi | HCT patients | 59 | CRP is inversely correlated with CL |
| Voriconazole | 2022 | Juan Chen | Immunocompromised Children | 91 | CRP is inversely correlated with CL |
| Voriconazole | 2022 | Zhiwen Jiang | patients with talaromycosis | 69 | CRP is inversely correlated with CL |
| Imatinib | 2005 | H Schmidli | chronic-phase chronic myeloid leukaemia | 371 | WBC is inversely correlated with CL |
| Ciclosporin | 2022 | Jing Ling | HSCT patients | 59 | CRP is positive correlated with CL |
| Linezolid | 2023 | SiChan Li | Post-operative Neurosurgical Patients | 22 | PCT is positive correlated with CL |
| Peficitinib | 2021 | Junko Toyoshima | rheumatoid arthritis (RA) | 989 | LYM is inversely correlated with CL |
| Darunavir | 2020 | Pier Giorgio Cojutti | HIV and COVID Patients | 30 | IL-6 is inversely correlated with CL/F |
| Tacrolimus | 2020 | Edouard Bonneville | liver transplant patients | 2 | CRP is postively correlated with trough concentration |
| Clozapine | 2016 | Gudrun Hefner | psychiatric patients | 33 | CRP is postively correlated with dose adjusted concentration |
| Risperidone | 2016 | Gudrun Hefner | psychiatric patients | 40 | CRP is postively correlated with dose adjusted concentration |
| Alprazolam | 2012 | Hadi Molanaei | hemodialysis patients | 26 | CRP is postively correlated with unconjugated alprazolam to 4-hydroxyalprazolam ratios |
| Alemtuzumab | 2020 | Senthil Velan Bhoopalan | haploidentical HCT | 13 | Absolute lymphocyte count is positively correlated with CL |
| Certolizumab | 2017 | Niels Vande Casteele | IBD CD | 2157 | CRP is positively correlated with median CL/F |
| Certolizumab | 2015 | Janet R Wade | IBD CD | 2157 | CRP is positively correlated with CL |
| Cetuximab | 2008 | Nathanael L Dirks | squamous cell carcinoma of the head and neck | 143 | WBC is positively correlated with CL |
| Golimumab | 2020 | Omoniyi J Adedokun | IBD UC | 1227 | CRP is positively correlated with CL |
| Golimumab | 2010 | Z H Xu | ankylosing spondylitis | 312 | Baseline CRP levels is positively correlated with CL |
| Golimumab | 2009 | Zhenhua Xu | psoriatic arthritis | 337 | CRP is positively correlated with CL |
| Infliximab | 2022 | Christian Primas | IBD patients | 41 | TNF-α and CRP are positively correlated with CL |
| Infliximab | 2021 | Ye Xiong | IBD CD | 78 | ESR is positively correlated with CL |
| infliximab | 2008 | Zhenhua Xu | ankylosing spondylitis | 274 | WBC is positively correlated with CL |
| JNJ-55920839 | 2020 | Zhenling Yao | Systemic Lupus Erythematosus | 26 | IFNα is positively correlated with CL |
| rATG | 2023 | Takuto Takahashi | HCT | 105 | CD4+ T cell is positively correlated with CL |
| Risankizumab | 2019 | Ahmed A Suleiman | Plaque Psoriasis | 1899 | hs-CRP is positively correlated with CL |
| Thymoglobulin | 2015 | Rick Admiraal | HCT | 280 | baseline lymphocyte count is positively correlated with CL |
| Tocilizumab | 2018 | Carla Bastida | RA patients | 35 | CRP is positively correlated with CL |
| Ustekinumab | 2022 | Omoniyi J Adedokun | IBD CD | 1673 | CRP is positively correlated with CL |

Supplementary table 3 Inflammation effects on medications of ICU and non-ICU

|  | ICU | Non-ICU |
| --- | --- | --- |
| Midazolam | CRP is negative correlated with CL[15] | CRP is negative correlated with CL [16] |
| Voriconazole | CRP is negative correlated with CL[17] | CRP is negative correlated with CL[18] |
| Imatinib | CRP is inversely correlated with CL[19] | WBC is inversely correlated with CL[20] |
| Lopinavir | Plasma concentrations are positively correlated with CRP[21] | Trough concentrations are positively correlated with CRP[22] |
| Tramadol | Tramadol metabolite is negatively correlated to Cholinesterase (ChE) activities[23] | Tramadol concentrations are positively correlated to IL-6 and CRP, while its metabolites are negatively correlated to these biomarkers[24] |
| Linezolid | Fibrinogen is positive correlated with CL[25] | PCT is positive correlated with CL[26] |
| Vancomycin | voncomycin plasma concentrations are negatively correlated to CRP[27] | the rates of change in the vancomycin dose/serum vancomycin concentration/estimated glomerular filtration rate ratio are positively correlated to CRP[28] |

Supplementary table 4 Promising inflammatory biomarkers for critically ill patients

| Biomarker Name | Type | Produced By | Function | Potentials/recommended reason |
| --- | --- | --- | --- | --- |
| IL-8 [29] | Cytokine | Macrophages, epithelial cells | Neutrophil chemotaxis, angiogenesis | Potential to be used predicted organ dysfunction |
| IL-10[30] | Cytokine | Macrophages, T-cells | immunosuppressive effects | IL-10 associated with organ functions |
| TNFα[31] | Cytokine | Macrophages, T-cells | Cell death, inflammation, immune response | Predictor of MODS |
| sCD14[32] | Glycoprotein | Monocytes, macrophages | Mediates response to bacterial LPS | potential marker of disease severity |
| sCD163[32] | Glycoprotein | Monocytes, macrophages | Anti-inflammatory signaling | potential marker of disease severity |
| Adrenomedullin (ADM)[33] | peptide hormone | Vascular Cells | vasopressor | Adrenomedullin (ADM) level is associated with organ function |
| von Willebrand Factor[34] | Glycoprotein | Endothelial cells, megakaryocytes | Blood clotting, platelet adhesion | Indicator of endothelial damage in COVID patients |
| BCL2[35] | Protein | Lymphocytes | Regulation of apoptosis | BCL2 on admission can predict MODS |
| CTproAVP[36] | Hormone | Hypothalamus | Water balance, vasoconstriction | correlate with disease severity |
| sTNFR-1[37] | Receptor | Various cell types | TNFα signaling modulation | linked strongly to severe AKI during respiratory illness |
| ANG2[38] | Glycoprotein | Endothelial cells | Angiogenesis, vascular permeability | severity, course and outcome of late onset ARDS |
| Macrophage migration inhibitory factor (MIF)[39] | protein | macrophage | host defense and stress response | associated with aggravated organ function and a significantly lower 28-day survival |
| soluble thrombomodulin [sTM][40] | protein | endothelium | biomarkers of endothelial injury | predictive biomarker for the development of AKI |
| soluble urokinase plasminogen activator receptor (suPAR)[41] | protein | immune cells | fibrinolytic pathway | Associated with organ dysfunction |
| circulating Dipeptidyl peptidase 3 (cDPP3)[42] | peptidase | Heart and lungs | degradation of various cardiovascular and endorphin mediators | cDPP3 level is associated with organ dysfunction |
| Serum resistin [43] | protein | Immune Cells | Insulin Resistance and inflammation | associated with renal failure and liver synthesis capacity |
| Retinol binding protein 4 (RBP4)[44] | protein | Liver and Adipose Tissue | primary carrier of retinol (vitamin A) | strong associations with hepatic and renal function |

Reference

1. Markanday, A., *Acute Phase Reactants in Infections: Evidence-Based Review and a Guide for Clinicians.* Open Forum Infect Dis, 2015. **2**(3): p. ofv098.

2. Foy, B.H., et al., *Human acute inflammatory recovery is defined by co-regulatory dynamics of white blood cell and platelet populations.* Nat Commun, 2022. **13**(1): p. 4705.

3. Kelly, M., J.M. Hwang, and P. Kubes, *Modulating leukocyte recruitment in inflammation.* J Allergy Clin Immunol, 2007. **120**(1): p. 3-10.

4. Valade, N., et al., *Thrombocytosis after trauma: incidence, aetiology, and clinical significance.* Br J Anaesth, 2005. **94**(1): p. 18-23.

5. Gabay, C. and I. Kushner, *Acute-phase proteins and other systemic responses to inflammation.* N Engl J Med, 1999. **340**(6): p. 448-54.

6. Sanz Codina, M. and M. Zeitlinger, *Biomarkers Predicting Tissue Pharmacokinetics of Antimicrobials in Sepsis: A Review.* Clin Pharmacokinet, 2022. **61**(5): p. 593-617.

7. Stanke-Labesque, F., et al., *Inflammation is a major regulator of drug metabolizing enzymes and transporters: Consequences for the personalization of drug treatment.* Pharmacol Ther, 2020. **215**: p. 107627.

8. Meisner, M., *Update on procalcitonin measurements.* Ann Lab Med, 2014. **34**(4): p. 263-73.

9. Zivkovic, A.R., et al., *Bedside-measurement of serum cholinesterase activity predicts patient morbidity and length of the intensive care unit stay following major traumatic injury.* Sci Rep, 2019. **9**(1): p. 10437.

10. Yang, Y., X. Yang, and J. Yang, *Cholinesterase level is a predictor of systemic inflammatory response syndrome and complications after cardiopulmonary bypass.* Ann Palliat Med, 2021. **10**(11): p. 11714-11720.

11. Dindo, D., et al., *Kinetics of D-dimer after general surgery.* Blood Coagul Fibrinolysis, 2009. **20**(5): p. 347-52.

12. Ceciliani, F. and C. Lecchi, *The Immune Functions of α(1) Acid Glycoprotein.* Curr Protein Pept Sci, 2019. **20**(6): p. 505-524.

13. Shimazui, T., et al., *Serum levels of interleukin-6 may predict organ dysfunction earlier than SOFA score.* Acute Med Surg, 2017. **4**(3): p. 255-261.

14. Maniar, R.N., et al., *What Is the Normal Trajectory of Interleukin-6 and C-reactive Protein in the Hours and Days Immediately After TKA?* Clin Orthop Relat Res, 2019. **477**(1): p. 41-46.

15. Smeets, T.J.L., et al., *Hyperinflammation Reduces Midazolam Metabolism in Critically Ill Adults with COVID-19.* Clin Pharmacokinet, 2022. **61**(7): p. 973-983.

16. Franken, L.G., et al., *Hypoalbuminaemia and decreased midazolam clearance in terminally ill adult patients, an inflammatory effect?* Br. J. Clin. Pharmacol., 2017. **83**(8): p. 1701-1712.

17. van den Born, D.A., et al., *Voriconazole exposure is influenced by inflammation: A population pharmacokinetic model.* Int J Antimicrob Agents, 2023. **61**(4): p. 106750.

18. Takahashi, T., et al., *Predictive Value of C-Reactive Protein and Albumin for Temporal Within-Individual Pharmacokinetic Variability of Voriconazole in Pediatric Patients Undergoing Hematopoietic Cell Transplantation.* J Clin Pharmacol, 2022. **62**(7): p. 855-862.

19. Bartelink, I.H., et al., *Elevated acute phase proteins affect pharmacokinetics in COVID-19 trials: Lessons from the CounterCOVID - imatinib study.* CPT Pharmacometrics Syst Pharmacol, 2021. **10**(12): p. 1497-1511.

20. Schmidli, H., et al., *Population pharmacokinetics of imatinib mesylate in patients with chronic-phase chronic myeloid leukaemia: results of a phase III study.* Br J Clin Pharmacol, 2005. **60**(1): p. 35-44.

21. Marzolini, C., et al., *Effect of Systemic Inflammatory Response to SARS-CoV-2 on Lopinavir and Hydroxychloroquine Plasma Concentrations.* Antimicrob Agents Chemother, 2020. **64**(9).

22. Schoergenhofer, C., et al., *Pharmacokinetics of Lopinavir and Ritonavir in Patients Hospitalized With Coronavirus Disease 2019 (COVID-19).* Ann Intern Med, 2020. **173**(8): p. 670-672.

23. Neskovic, N., et al., *Different Pharmacokinetics of Tramadol, O-Demethyltramadol and N-Demethyltramadol in Postoperative Surgical Patients From Those Observed in Medical Patients.* Front Pharmacol, 2021. **12**: p. 656748.

24. Tanaka, H., et al., *Impact of CYP genotype and inflammatory markers on the plasma concentrations of tramadol and its demethylated metabolites and drug tolerability in cancer patients.* Eur J Clin Pharmacol, 2018. **74**(11): p. 1461-1469.

25. Taubert, M., et al., *Predictors of Inadequate Linezolid Concentrations after Standard Dosing in Critically Ill Patients.* Antimicrob Agents Chemother, 2016. **60**(9): p. 5254-5261.

26. Li, S., et al., *Population Pharmacokinetics and Dosing Regimen Optimization of Linezolid in Cerebrospinal Fluid and Plasma of Post-operative Neurosurgical Patients.* J Pharm Sci, 2023. **112**(3): p. 884-892.

27. Cousin, V.L., et al., *Role of fluid status markers as risk factors for suboptimal vancomycin concentration during continuous infusion in neonates: an observational study.* Eur J Pediatr, 2022. **181**(8): p. 2935-2942.

28. Okamoto, G., et al., *Pharmacokinetic variability of vancomycin in patients with nosocomial meningitis.* J Clin Pharm Ther, 2022. **47**(11): p. 1752-1758.

29. Ishikawa, S., et al., *Risk prediction of biomarkers for early multiple organ dysfunction in critically ill patients.* BMC Emerg Med, 2021. **21**(1): p. 132.

30. Jones, M.A., et al., *Plasma interleukin responses as predictors of outcome stratification in patients after major trauma: a prospective observational two centre study.* Front Immunol, 2023. **14**: p. 1276171.

31. Trautinger, F., et al., *Respiratory burst capability of polymorphonuclear neutrophils and TNF-alpha serum levels in relationship to the development of septic syndrome in critically ill patients.* J Leukoc Biol, 1991. **49**(5): p. 449-54.

32. Attia, H., M. El Nagdy, and R.M. Abdel Halim, *Preliminary Study of sCD14 and sCD163 as Predictors of Disease Severity and ICU Admission in COVID-19: Relation to Hematological Parameters, Blood Morphological Changes and Inflammatory Biomarkers.* Mediterr J Hematol Infect Dis, 2023. **15**(1): p. e2023046.

33. Mebazaa, A., et al., *Circulating adrenomedullin estimates survival and reversibility of organ failure in sepsis: the prospective observational multinational Adrenomedullin and Outcome in Sepsis and Septic Shock-1 (AdrenOSS-1) study.* Crit Care, 2018. **22**(1): p. 354.

34. Joly, B.S., et al., *Imbalance of von Willebrand factor and ADAMTS13 axis is rather a biomarker of strong inflammation and endothelial damage than a cause of thrombotic process in critically ill COVID-19 patients.* J Thromb Haemost, 2021. **19**(9): p. 2193-2198.

35. El Shazly, A.N., et al., *Evaluation of BCL2 and TNFα as mRNA biomarkers for monitoring the immune response in critically ill children.* Ann Med Surg (Lond), 2018. **36**: p. 122-128.

36. Koch, A., et al., *Clinical relevance of copeptin plasma levels as a biomarker of disease severity and mortality in critically ill patients.* J Clin Lab Anal, 2018. **32**(9): p. e22614.

37. Sathe, N.A., et al., *Biomarker Signatures of Severe Acute Kidney Injury in a Critically Ill Cohort of COVID-19 and Non-COVID-19 Acute Respiratory Illness.* Crit Care Explor, 2023. **5**(7): p. e0945.

38. Hoeboer, S.H., et al., *Serial inflammatory biomarkers of the severity, course and outcome of late onset acute respiratory distress syndrome in critically ill patients with or at risk for the syndrome after new-onset fever.* Biomark Med, 2015. **9**(6): p. 605-16.

39. Bleilevens, C., et al., *Macrophage Migration Inhibitory Factor (MIF) Plasma Concentration in Critically Ill COVID-19 Patients: A Prospective Observational Study.* Diagnostics (Basel), 2021. **11**(2).

40. Katayama, S., et al., *Markers of acute kidney injury in patients with sepsis: the role of soluble thrombomodulin.* Crit Care, 2017. **21**(1): p. 229.

41. Klausen, H.H., et al., *How inflammation underlies physical and organ function in acutely admitted older medical patients.* Mech Ageing Dev, 2017. **164**: p. 67-75.

42. Blet, A., et al., *Monitoring circulating dipeptidyl peptidase 3 (DPP3) predicts improvement of organ failure and survival in sepsis: a prospective observational multinational study.* Crit Care, 2021. **25**(1): p. 61.

43. Koch, A., et al., *Serum resistin levels in critically ill patients are associated with inflammation, organ dysfunction and metabolism and may predict survival of non-septic patients.* Crit Care, 2009. **13**(3): p. R95.

44. Koch, A., et al., *Circulating retinol binding protein 4 in critically ill patients before specific treatment: prognostic impact and correlation with organ function, metabolism and inflammation.* Crit Care, 2010. **14**(5): p. R179.
