# Supplementary material for: The relation between inflammatory biomarkers and drug pharmacokinetics in the critically ill patients: a scoping review
Source: Crit Care. 2024 Nov 19;28:376. doi: 10.1186/s13054-024-05150-4 (PMC11577668; doi:10.1186/s13054-024-05150-4)
Supplement: Supplementary file 2 — Additional file 2. [file 13054_2024_5150_MOESM2_ESM.docx]

| **Database searched** | **Platform** | **Years of coverage** | **Records** | **Records after duplicates removed** |
| --- | --- | --- | --- | --- |
| Medline ALL | Ovid | 1946 - Present | 990 | 988 |
| Embase | Embase.com | 1971 - Present | 3954 | 3180 |
| Web of Science Core Collection* | Web of Knowledge | 1975 - Present | 1185 | 554 |
| Cochrane Central Register of Controlled Trials | Wiley | 1992 - Present | 192 | 57 |
| **Total** | | | **6321** | **4779** |

*Science Citation Index Expanded (1975-present) ; Social Sciences Citation Index (1975-present) ; Arts & Humanities Citation Index (1975-present) ; Conference Proceedings Citation Index- Science (1990-present) ; Conference Proceedings Citation Index- Social Science & Humanities (1990-present) ; Emerging Sources Citation Index (2005-present)

No other database limits were used than those specified in the search strategies

**medline**

(Pharmacokinetics / OR Pharmacokinetics .fs. OR Pharmacogenomic Variants/ OR Pharmacogenetics/ OR (pharmacokinetic* OR pharmaco-kinetic* OR pharmacodynamic* OR pharmaco-dynamic* OR pharmacogenomic* OR pharmaco-genomic*).ab,ti,kw. OR ((drug* OR agent* OR pharmac*) ADJ3 (absorpt* OR accumulat* OR activat* OR adsorpt* OR bioavailability* OR clearance* OR dialysability* OR diffus* OR disposit* OR distribut* OR eliminat* OR excret* OR half-life* OR inactivat* OR metabolism* OR penetrat* OR release* OR retent*)).ti.) AND (Inflammation/ OR Sepsis/ OR C-Reactive Protein/ OR Interleukin-6/ OR Interferons / OR Leukocyte/ OR Procalcitonin/ OR Neutrophils / OR Lymphocytes / OR Fibrin Fibrinogen Degradation Products / OR Blood Sedimentation / OR Interleukin-1/ OR Interleukin-1beta/ OR Interleukin-8/ OR Tumor Necrosis Factor-alpha/ OR Serum Amyloid A Protein/ OR Lactic Acid/ OR Ferritins / OR (inflammat* OR hyperinflammat* OR sepsis* OR septic* OR Ferritin* OR Lactic-acid OR Lactate* OR (serum* ADJ3 amyloid-A) OR (tumor ADJ3 necrosis ADJ3 factor*) OR Tnf* OR (erythrocyte* ADJ3 sedimentat*) OR C-Reactive-Protein* OR CRP OR Interleukin-6 OR IL6 OR IL-6 OR Interleukin-8 OR IL8 OR IL-8 OR Interleukin-1* OR IL1* OR IL-1* OR Interferon* OR IFN OR PCT OR Leukocyte* OR WBC OR White-Blood-Cell* OR Procalcitonin* OR Pro-Calcitonin* OR Calcitonin-1 OR PCT OR Neutrophil* OR lymphocyte* OR D-dimer* OR fibrin-fragment-D).ab,ti,kw.) AND (Intensive Care Units/ OR Critical Care/ OR Critical Illness/ OR (intensive-care* OR icu OR (critical* ADJ3 ill*)).ab,ti,kw.) NOT (exp animals/ NOT humans/) AND english.la.

**embase**

(pharmacokinetics/de OR pharmacodynamics/de OR pharmacodynamics/exp/mj OR pharmacokinetics/exp/mj OR 'pharmacogenetic variant'/de OR pharmacogenomics/de OR 'pharmacokinetic parameters'/de OR 'pharmacokinetic parameters'/exp/mj OR (pharmacokinetic* OR pharmaco-kinetic* OR pharmacodynamic* OR pharmaco-dynamic* OR pharmacogenomic* OR pharmaco-genomic*):ab,ti,kw OR ((drug* OR agent* OR pharmac*) NEAR/3 (absorpt* OR accumulat* OR activat* OR adsorpt* OR bioavailability* OR clearance* OR dialysability* OR diffus* OR disposit* OR distribut* OR eliminat* OR excret* OR half-life* OR inactivat* OR metabolism* OR penetrat* OR release* OR retent*)):ti) AND (inflammation/de OR 'inflammatory marker'/de OR sepsis/exp OR 'C reactive protein'/de OR 'interleukin 6'/de OR interferon/exp OR Leukocyte/exp OR procalcitonin/de OR neutrophil/de OR lymphocyte/exp OR 'D dimer'/de OR 'fibrin fragment d'/de OR 'erythrocyte sedimentation rate'/de OR 'interleukin 1'/de OR 'interleukin 1beta'/de OR 'interleukin 8'/de OR 'tumor necrosis factor'/de OR 'serum amyloid A'/de OR 'lactic acid'/de OR ferritin/de OR (inflammat* OR hyperinflammat* OR sepsis* OR septic* OR Ferritin* OR Lactic-acid OR Lactate* OR (serum* NEAR/3 amyloid-A) OR (tumor NEAR/3 necrosis NEAR/3 factor*) OR Tnf* OR (erythrocyte* NEAR/3 sedimentat*) OR C-Reactive-Protein* OR CRP OR Interleukin-6 OR IL6 OR IL-6 OR Interleukin-8 OR IL8 OR IL-8 OR Interleukin-1* OR IL1* OR IL-1* OR Interferon* OR IFN OR PCT OR Leukocyte* OR WBC OR White-Blood-Cell* OR Procalcitonin* OR Pro-Calcitonin* OR Calcitonin-1 OR PCT OR Neutrophil* OR lymphocyte* OR D-dimer* OR fibrin-fragment-D):Ab,ti,kw) AND ('intensive care unit'/exp OR 'intensive care'/de OR 'critically ill patient'/de OR 'critical illness'/de OR (intensive-care* OR icu OR (critical* NEAR/3 ill*)):Ab,ti,kw) NOT [conference abstract]/lim NOT ([animals]/lim NOT [humans]/lim) AND [english]/lim

**Web of science**

TS=(((pharmacokinetic* OR pharmaco-kinetic* OR pharmacodynamic* OR pharmaco-dynamic* OR pharmacogenomic* OR pharmaco-genomic*)) AND ((inflammat* OR hyperinflammat* OR sepsis* OR septic* OR Ferritin* OR Lactic-acid OR Lactate* OR (serum* NEAR/2 amyloid-A) OR (tumor NEAR/2 necrosis NEAR/2 factor*) OR Tnf* OR (erythrocyte* NEAR/2 sedimentat*) OR C-Reactive-Protein* OR CRP OR Interleukin-6 OR IL6 OR IL-6 OR Interleukin-8 OR IL8 OR IL-8 OR Interleukin-1* OR IL1* OR IL-1* OR Interferon* OR IFN OR PCT OR Leukocyte* OR WBC OR White-Blood-Cell* OR Procalcitonin* OR Pro-Calcitonin* OR Calcitonin-1 OR PCT OR Neutrophil* OR lymphocyte* OR D-dimer* OR fibrin-fragment-D)) AND ((intensive-care* OR icu OR (critical* NEAR/2 ill*)))) AND DT=(article) AND LA=(english)

**Cochrane**

((pharmacokinetic* OR pharmaco-kinetic* OR pharmacodynamic* OR pharmaco-dynamic* OR pharmacogenomic* OR pharmaco-genomic*):ab,ti,kw) AND ((inflammat* OR hyperinflammat* OR sepsis* OR septic*):Ab,ti,kw) AND ((intensive-care* OR icu OR (critical* NEAR/3 ill*)):Ab,ti,kw) NOT ("conference abstract":kw OR Trial registry record:pt)
